# Selective Activation of Cholecystokinin-Expressing GABA (CCK-GABA) Neurons Enhances Memory and Cognition

**DOI:** 10.1523/ENEURO.0360-18.2019

**Published:** 2019-02-27

**Authors:** Paul D. Whissell, Jee Yoon Bang, Ikram Khan, Yu-Feng Xie, Gustavo M. Parfitt, Martine Grenon, Nicholas W. Plummer, Patricia Jensen, Robert P. Bonin, Jun Chul Kim

**Affiliations:** 1 Psychology; 2 Cell and Systems Biology, University of Toronto, Toronto, Ontario, Canada M5S 3G3; 3 Leslie Dan Faculty of Pharmacy, University of Toronto, Toronto, Ontario, M5S 3M2, Canada; 4 Neurobiology Laboratory, National Institute of Environmental Health Sciences, National Institutes of Health, Department of Health and Human Services, Research Triangle Park, NC 27709

**Keywords:** anxiety, cholecystokinin, cognition, GABA, memory, perisomatic

## Abstract

Cholecystokinin-expressing GABAergic (CCK-GABA) neurons are perisomatic inhibitory cells that have been argued to regulate emotion and sculpt the network oscillations associated with cognition. However, no study has selectively manipulated CCK-GABA neuron activity during behavior in freely-moving animals. To explore the behavioral effects of activating CCK-GABA neurons on emotion and cognition, we utilized a novel intersectional genetic mouse model coupled with a chemogenetic approach. Specifically, we generated triple transgenic *CCK-Cre;Dlx5/6-Flpe;RC::FL-hM3Dq* (CCK-GABA/hM3Dq) mice that expressed the synthetic excitatory hM3Dq receptor in CCK-GABA neurons. Results showed that clozapine-N-oxide (CNO)-mediated activation of CCK-GABA neurons did not alter open field (OF) or tail suspension (TS) performance and only slightly increased anxiety in the elevated plus maze (EPM). Although CNO treatment had only modestly affected emotional behavior, it significantly enhanced multiple cognitive and memory behaviors including social recognition, contextual fear conditioning, contextual discrimination, object recognition, and problem-solving in the puzzle box. Collectively, these findings suggest that systemic activation of CCK-GABA neurons minimally affects emotion but significantly enhances cognition and memory. Our results imply that CCK-GABA neurons are more functionally diverse than originally expected and could serve as a potential therapeutic target for the treatment of cognitive/memory disorders.

## Significance Statement

Cholecystokinin-expressing GABAergic (CCK-GABA) neurons are thought to play an important role in pathologies such as schizophrenia, but their contributions to behavior in healthy states are poorly understood. Here we report a novel method for selectively targeting CCK-GABA neurons and manipulating their activity during behavioral tasks. Our data demonstrate that activating CCK-GABA neurons subtly affects emotional behavior but surprisingly enhances multiple memory and cognitive processes.

## Introduction

The inhibitory neurotransmitter gamma-aminobutyric acid (GABA) critically regulates information processing by modifying neuronal excitability and synaptic plasticity (Wigström and Gustafsson, 1983; Sloviter, 1991). GABAergic transmission contributes to multiple behaviors, including anxiety, fear, pain, memory, olfaction and social interaction (Enna and McCarson, 2006; Makkar et al., 2010; Schmidt et al., 2014). Dysregulation of GABAergic transmission is a defining feature of many pathologies, including schizophrenia, chronic pain, depression, and Alzheimer’s disease (Rissman et al., 2003; Enna and McCarson, 2006; Luscher et al., 2011; Taylor and Tso, 2015). GABAergic signaling is orchestrated by interneurons, a heterogenous cell population composed of many subtypes differing in morphologic, electrophysiological and neurochemical properties as well as connectivity patterns (Kepecs and Fishell, 2014). There is widely believed to be a “division of labor” among these many interneurons, wherein distinct subtypes are specialized for certain functions (Kepecs and Fishell, 2014).

Interneurons expressing the neuropeptide cholecystokinin (CCK; termed CCK-GABA neurons) are recognized for their characteristic axonal projections, which ramify extensively around the perisomatic regions (i.e., cell body, proximal dendrites, and axon initial segments) of postsynaptic pyramidal cells. It has been hypothesized that CCK-GABA neurons are functionally specialized for the regulation of emotional behaviors such as mood, anxiety and fear (Freund, 2003). Notably, CCK-GABA neurons express several receptor subtypes involved in controlling emotional behavior, including 5-hydroxytryptamine type 3 receptors (5-HT3) and cannabinoid type 1 (CB1) receptors (Morales and Bloom, 1997; Marsicano and Lutz, 1999). Additionally, the postsynaptic targets of CCK-GABA neurons are enriched with α2-containing GABA_A_ receptors, which regulate anxiety behaviors and the effects of anxiolytic drugs (Freund, 2003). However, compelling evidence for the role of CCK-GABA neurons in emotional behaviors has proven elusive, with studies producing conflicting findings (Truitt et al., 2009; Brown et al., 2014; Schmidt et al., 2014; Bowers and Ressler, 2015).

An alternative and underappreciated possibility is that CCK-GABA cells play an influential role in cognitive and memory processes. Several lines of evidence support this theory. Firstly, CCK-GABA cells are comparatively abundant (20–30% of all GABA neurons) in brain regions involved in cognitive/memory processes, such as the medial prefrontal cortex, hippocampus and ventrolateral temporal cortex (Whissell et al., 2015). Second, CCK-GABA neurons are thought to orchestrate the theta frequency network oscillations associated with cognitive processes as they elicit slow, asynchronous inhibitory events in their targets (Curley and Lewis, 2012; Nagode et al., 2014). Third, the presynaptic nerve terminals of CCK-GABA neurons are enriched with CB1 receptors (Katona et al., 1999), which have been implicated in cognition and memory as well as the neuroplasticity that accompanies these processes (Carlson et al., 2002). Collectively, these remarkable features suggest CCK-GABA neurons are well-positioned to regulate cognition and memory.

To gain better insight into the behavioral effects of enhanced CCK-GABA neuron activity, we selectively activated these cells during tests of emotion, cognition and memory by using an intersectional genetic approach (Sciolino et al., 2016) coupled with the chemogenetic technique (Armbruster et al., 2007). In our novel transgenic line, the CCK-GABA/hM3Dq mouse, CCK-GABA neurons express the synthetic excitatory Gq-coupled hM3Dq receptor which can be selectively activated by the synthetic ligand, clozapine-N-oxide (CNO). To determine the behavioral effects of CCK-GABA neuron activation, we studied different emotional and cognitive behaviors in CCK-GABA/hM3Dq mice given CNO or vehicle injection. We observed that CCK-GABA neuron activation minimally affected emotional behavior but significantly enhanced memory and cognition.

## Materials and Methods

### Animals

To generate triple transgenic *CCK-Cre;Dlx5/6-Flpe;RC::FL-hM3Dq* mice (termed CCK-GABA/hM3Dq^+^ mice), homozygous CCK-ires-Cre mice (C57BL/6 genetic background, B6N.Cg-*Cck^tm1.1(cre)Zjh^*/J, JAX#019021) were first crossed with homozygous *RC::FL-hM3Dq* mice (C57BL/6 genetic background; Sciolino et al., 2016). Subsequently, double transgenic *CCK-Cre;RC::FL-hM3Dq* mice were crossed with *Dlx5/Dlx6-FLPe* mice (FVB/NC genetic background, Tg(mI56i-FLPe)39Fsh/J, JAX#010815) to obtain triple transgenic mice having all three alleles (Cre, hM3Dq and Flpe). Double transgenic mice having Cre and hM3Dq alleles but not the Flpe allele were referred to as CCK-GABA/hM3Dq^-^ mice and used as age-matched littermate controls. Both male and female mice were used in experiments. Mice were group housed with ad libitum access to food and water in a temperature-controlled room on a 12/12 h light/dark cycle. All behavioral testing occurred during the light phase. Animals underwent multiple behavioral tests in the following order (starting with the least aversive test and proceeding to the most aversive test): open field (OF) test, elevated plus maze (EPM) test, novel object recognition test, puzzle box test, social interaction test, and fear conditioning test. As the fear conditioning test resulted in long-term changes in animal behavior, a separate population of naïve animals was used for the tail suspension (TS) test. In all behavioral assays but the novel object recognition test, a between-subject design was used wherein animals were randomly assigned to either the vehicle injection condition or drug injection condition before the experiment began. In all branches of the novel object recognition test, which followed a within-subject design, animals were tested twice and received each injection. Experimental procedures were in accordance with the guidelines of the Canadian Council on Animal Care and the local Animal Care Committee at University of Toronto.

### Immunohistochemistry and image acquisition

Triple transgenic mice three to six months old were anesthetized with avertin and underwent transcardial perfusion with 0.1 M PBS (pH 7.4) followed by 4% paraformaldehyde (PFA) in PBS. Extracted brains were placed into 4% PFA at 4°C for 24 h and then transferred into a PBS solution containing 30% sucrose at 4°C for 48 h. Afterward, brains were cut into 40 µM sections using a cryostat (CM1520; Leica) maintained at –20°C. From each brain, 10 sections were obtained in the area of the dorsal hippocampus (bregma = –1.34 to –1.94 mm).

In wide field microscopy experiments, tissue sections were rinsed with 0.1 M PBS and blocked with 5% normal donkey serum in 0.1% Triton X-100 PBS (PBS-T) for 1 h at room temperature. Sections were then incubated with chicken polyclonal anti-green fluorescent protein (GFP; 1:1000; ab13970; Abcam) and rabbit polyclonal anti-mCherry (1:1000; ab167453; Abcam) primary antibodies in PBS-T for 48 h at 4°C. Thereafter, sections were rinsed with PBS-T and incubated with Alexa Fluor 488-conjugated donkey anti-chicken (1:1000; 703545145; Jackson ImmunoResearch) and Alexa Fluor 594-conjugated donkey anti-rabbit (1:1000; 715515152; Jackson ImmunoResearch) secondary antibodies in PBS-T for 2 h at room temperature. Sections were then rinsed with PBS-T and mounted on Superfrost Plus slides (Fisher Scientific) and coverslipped with Aquamount (Polysciences Inc.). In experiments examining colocalization of mCherry with CCK and glutamic acid decarboxylase 67 (GAD67), sections were stained using a different procedure. During primary antibody staining, sections were incubated with goat anti-mCherry antibody (1:1000; AB0040; Sicgen), mouse anti-GAD67 antibody (1:1000; Mab5406; Millipore) and rabbit polyclonal anti-CCK-8 antibody (1:1000; C2581; Sigma-Aldrich) in PBS-T for 48 h at 4°C. In secondary antibody staining, sections were incubated in PBS-T with Alexa Fluor 594-conjugated donkey anti-goat antibody (1:1000; 705515147; Jackson ImmunoResearch), DyLight 488-conjugated donkey anti-mouse antibody (1:1000; 715485150; Jackson ImmunoResearch), and Alexa Fluor 405-conjugated donkey anti-rabbit antibody (1:1000; ab175651; Abcam) for 2 h at room temperature.

For cell counting experiments, images of brain sections were generated using an FSX100 fluorescent microscope (Olympus). Alexa Fluor 488 and 594 signals were captured using a U-MWIBA3 filter cube (Ex460-495, Em510-550, DM505) for Alexa Fluor 488 and a U-MWIG3 filter cube (Ex530-550, Em575IF, DM570) for Alexa Fluor 594. In acquired images, regions of interest in the hippocampus and prefrontal cortex were delineated manually according to area definitions established by Paxinos and Franklin (2012). Automated cell counting of GFP-labeled and mCherry-labeled cells in delineated brain regions was performed using cellSens 1.7 software (Olympus). For each animal, we calculated the relative abundance of mCherry-labeled cells for a region of interest (= mCherry-labeled cells/[mCherry-labeled cells + GFP-labeled cells]) by averaging all values for that region across all sections. For colocalization experiments, images were captured through a Quorum spinning disk confocal microscope (Zeiss) using a 20× objective lens and were analyzed with Volocity Software (PerkinElmer). Alexa Fluor 405, 488, and 594 (secondary antibody signals) were excited with the 405-, 491-, and 561-nm laser, respectively.

### Drugs

CNO was obtained from the NIH as a part of the Rapid Access to Investigative Drug Program funded by the National Institute of Neurologic Disorders and Stroke (NINDS). CNO powder was dissolved in 20% dimethylsulfoxide (DMSO)/saline to prepare a stock solution of 3 mM. In behavioral experiments, mice were weighed daily before being randomly assigned to either CNO or vehicle treatment groups. Unless otherwise stated, all drug injections were given in the intraperitoneal cavity ∼10 min before each test. Mice in the CNO group received a 3 mg/kg injection of CNO whereas mice in the vehicle group received a DMSO/saline injection. The experimenters giving the injections and testing the mice were blinded to the drug condition. To ensure that CNO injection of an animal in one behavioral test did not confound the performance of that animal in another subsequent behavioral test, we made sure that different behavioral tests were separated by at least 48 h. In electrophysiology experiments, a concentration of 5 µM CNO was used.

### Electrophysiology, tissue preparation

Male CCK-GABA/hM3Dq+ and CCK-GABA/hM3Dq^-^ mice two to three months old were used. Mice were deeply anesthetized with chloral hydrate (400 mg/kg; i.p.) followed by transcardial perfusion with ice-cold solution containing: 50 mM sucrose, 92 mM NaCl, 15 mM D-glucose, 26 mM NaHCO_3_, 5 mM KCl, 1.25 mM NaH_2_PO_4_, 0.5 mM CaCl_2_, mM 7 MgSO_4_, and 1 mM kynurenic acid that was oxygenated with 95% O_2_/5% CO_2_. The brain was removed and transverse hippocampus slices (300 µm) were cut with a vibratome (Leica VT-1200S) and thereafter incubated at room temperature in artificial CSF (aCSF) containing: 124 mM NaCl, 3 mM KCl, 1.25 mM NaH_2_PO_4_, 1.3 mM MgCl_2_, 2.6 mM CaCl_2_, 26 mM NaHCO_3_, and 10 mM D-glucose (300–310 mOsm) that was oxygenated with 95% O_2_/5% CO_2_.

### Electrophysiology, recording

Hippocampal slices were perfused with aCSF at 2–3 ml/min. Cells were visually identified using a microscope (BX-51W1; Olympus) fitted with IR-DIC and X-Cite LED120 fluorescence illumination (Excelitas Technologies) filtered with FITC and TRITC cubes (Olympus) for detection of mCherry expression in CCK-GABA/hM3Dq^+^ cells. The recording pipette had a resistance of 4–6 MΩ. For current-clamp experiments the pipette was filled with intracellular solution containing: 132.5 mM K-gluconate, 17.5 mM KCl, 10 mM HEPES, 0.2 mM EGTA, 2 mM Mg-ATP, and 0.3 mM GTP (pH 7.25, 290 mOsm). For voltage-clamp experiments where miniature IPSCs (mIPSCs) were recorded, the intracellular solution contained: 140 mM CsCl, 10 mM HEPES, 11 mM EGTA, 1 mM CaCl_2_, 2 mM MgCl_2_, 2 mM tetraethylammonium chloride, and 4 mM Mg-ATP (pH 7.25, 290 mOsm). Neurons were held at –60 mV, and mIPSCs were recorded with TTX (0.2 µm), APV (50 µM), and CNQX (10 µm) added to the aCSF. Spontaneous action potential frequency and resting membrane potential were recorded in current clamp mode. TTX (0.2 µm) was added to the aCSF for determination of resting membrane potential. Electrophysiological recordings were amplified with Multiclamp 700A (Molecular Devices), filtered at 2 kHz, sampled 50 kHz, and analyzed offline with Clampfit 10 (Molecular Devices). Stable 3-min recordings of mIPSCs were selected for measuring mIPSC amplitude and frequency. Action potential frequency was measured by averaging the number of spontaneous action potentials over a 5-min window at baseline or after bath application of CNO.

### OF test

Animals were placed in an OF box (50 cm L × 50 cm W × 20 cm H) for 10 min. The 35 × 35 region in the center of the arena was defined as the center whereas the remainder was defined as the periphery zone. Automated scoring of zone time and distance traveled was coordinated by an experimenter blind to condition using Ethovision XT 11.5 software.

### EPM

The 5 min test was conducted in a plus-shaped maze composed of two “open” arms without walls (30 cm L × 5 cm W) and two “closed” arms (30 cm L × 5 cm W) enclosed by walls (10 cm H) arranged around a center zone (5 cm L × 5 cm W). Automated scoring of arm time, arm entries and distance traveled was coordinated by an experimenter blind to condition using Ethovision XT 11.5 software.

### TS test

The test took place in a tall chamber (20 cm L × 50 cm W × 50 cm H). To prevent bending of the mouse’s tail during the test, a lightweight plastic cylinder (8 cm long, 2 g in weight) was placed around the tail base. Next, a length of adhesive tape (20 cm) was gently but firmly wrapped around the tail 2 cm from the tail tip. This length of tape was then fixed to the roof of the chamber, suspending the mouse in the air. The test lasted 6 min and the time that the mouse spent immobile was scored by an experimenter blind to condition.

### Social interaction test

The three-chambered sociability test was conducted under red light (3 lux) in a clear Plexiglas box with three compartments (40 cm L × 20 cm W × 40 cm H) separated by walls. The center-facing walls of the left and right chambers contained an opening (5 cm W × 40 cm H) that allowed passage into the center chamber. Each of the left and right chambers contained a wire cage (11 cm D × 11 cm H, 1 cm bar spacing). Before testing, mice were habituated to the apparatus for 10 min (habituation trial). Afterward, the test mouse was placed in a holding cage while a novel juvenile mouse (23–35 d of age) unfamiliar to the test mouse was placed inside one of the wire cages. The test mouse was returned to the apparatus and the time spent interacting with the stranger mouse was recorded (sociability trial, 10 min). Afterward, both mice were removed and placed in separate holding cages while the apparatus was cleaned with 70% ethanol. The mouse from the sociability trial, now familiar to the test mouse, was then returned to one of the wire cages. A second novel mouse, completely unfamiliar to the test mouse, was then added in the opposite wire cage (social recognition trial, 10 min). The test mouse was then returned to the apparatus and interaction time with the “familiar” and “novel” mouse was measured manually by an experimenter blind to condition using a keystroke function in Ethovision XT 11.5 software.

### Fear conditioning and contextual fear discrimination

A conditioning chamber (20 cm L × 20 cm W × 30 cm H) with a shock grid floor consisting of stainless-steel bars (2 cm apart, diameter = 2 mm) was used (Coulbourn Instruments). In every trial, the percentage of time spent “freezing” (absence of movement except respiration) was scored using FreezeFrame software (Coulbourn Instruments). The experiment took place over 3 d. On day 1, mice acquired a fear conditioning response in the training context (termed context A) by receiving 5 tone-shock pairings over 480 s. Every acquisition trial began with a 180-s habituation period wherein the mouse explored the chamber freely. At 160 s into the trial, a tone (70 dB, duration = 20 s) was delivered. At 178 s into the trial, a mild footshock (0.7 mA, duration = 2 s) was delivered and co-terminated with the tone. A further four tone-signaled footshocks were delivered at 240, 300, 360, and 420 s. After 480 s, the mouse was removed from the chamber and returned to their home cage for 24 h. On day 2 in the morning (9–12 A.M.), contextual fear memory was measured by re-exposing mice to context A. On day 2 in the afternoon (12–3 P.M.), contextual discrimination was measured by placing mice in a second context (context A’) that was similar to context A but differed subtly in visual cues (lighting/wallpaper). Contextual discrimination was measured using the discrimination index (A’ vs A), which was defined as: (Freezing in A on Day 2) / (Freezing in A’ on Day 2 + Freezing in A on Day 2). Chambers were cleaned with 70% ethanol between exposures. On day 3, cued fear conditioning was measured by re-exposing the mice to the tone in a novel chamber (context B) that differed significantly from context A in olfactory cues, visual cues and overall volume. In the cued fear conditioning trial, mice were given 180 s to explore the chamber before the tone was delivered for 300 s.

### Novel object recognition

Mice were habituated for 15 min to the testing chamber (20 cm L × 20 cm W × 20 cm H) before the protocol began. On the following day, each mouse was placed in the chamber for 15 min with two identical objects (FO1 + FO2; training phase). Afterward, the animal was either placed in a holding cage for 60 min (short-term version) or returned to their home cage for 24 h (long-term version; delay phase). During this time, the chamber was cleaned with 70% ethanol and one of the objects (FO2) was replaced with a novel object (NO). The mouse was then placed back in the chamber (Testing Phase), and the time that it spent interacting with each object was measured. Novel object preference (%) was calculated as = NO interaction/(FO1 interaction + NO interaction) whereas total interaction time was calculated as = FO1 interaction + NO interaction. A within-subject design was used wherein all animals were exposed to all four conditions (vehicle/post-training, vehicle/pre-training, CNO/post-training, and CNO/pre-testing) over a period of 8 d in a counterbalanced order using different sets of objects. In the post-training injection condition, CNO injection was given immediately following training. In the pre-testing injection condition, CNO injection was given 15 min before the testing phase. Automated scoring was coordinated by an experimenter blind to condition using Ethovision XT 11.5 software. Animals with an interaction time of <3 s were excluded from analysis.

### Puzzle box

The box was a two-chambered apparatus consisting of a start area (58 × 28 × 28 cm^3^) that was brightly illuminated and a goal area (14 × 28 × 28 cm^3^) that was enclosed by walls and filled with bedding. Initially, the start area was connected to the goal area via a doorway and underpass. During the task, the passage to the goal area was obscured by obstacles of increasing difficulty. The task took place over 3 d with three trials per day (D1: trial 1–3, D2: trial 4–6, D3: trial 7–9). On the first day of testing, animals were placed in either the vehicle injection group or CNO injection group and received the same injection once per day, 10 min before testing began. On trial 1 (T1), the goal area was accessible through the doorway and underpass. On T2, the doorway was blocked and the goal area was only accessible through the underpass (underpass task). This process was repeated for T3 and T4. On T5, the doorway remained blocked and the underpass was filled with corncob bedding. To enter the goal area, the mice would have to dig through this bedding (dig task). This process was repeated for T6 and T7. During T8, the doorway remained blocked and the underpass was instead filled with a cardboard plug (plug task). To enter the goal area, mice would have to remove/rearrange this plug. This process was repeated in T9. As T2, T5, and T8 involved the introduction of a novel problem (underpass, dig task, and plug task, respectively) analysis focused on these trials. In between trials, mice were placed in a holding cage while the chamber was cleaned with 70% ethanol. All scoring was done manually by an experimenter blind to condition. If a mouse failed to solve the task within 300 s, the trial was terminated and a time of 300 s was assigned.

### Statistical analysis

In all cases, we used a randomized experimental design. All analysis was performed using GraphPad Prism 6.0 for Windows. For electrophysiological experiments, the Wilcoxon matched pairs signed rank test was used to compare action potential frequency before and after application of CNO, while Student’s *t* tests (paired) were performed with significance set at *p* = 0.05 for all other analyses. In behavioral experiments, *t* tests were generally used but two-way ANOVA was employed in several cases. In OF and puzzle box experiments, two-way ANOVA was used with drug as a between-subjects factor and time as a within-subjects factor. In OF and EPM experiments, two-way ANOVA was used with drug as a between-subjects factor and compartment as a within-subjects factor. *Post hoc* analysis for two-way ANOVA was performed using Sidak’s multiple comparison test with significance set at *p* = 0.05. In all experiments, cases with a score of more than two absolute standard deviation units from the mean were classified as outliers and excluded from analysis. This criterion resulted in the exclusion of between 0 and 1 animal per experiment. All figures present data as mean ± SEM.

## Results

### Selective targeting of CCK-GABA neurons in the CCK-GABA/hM3Dq mouse line

**Figure 1.**
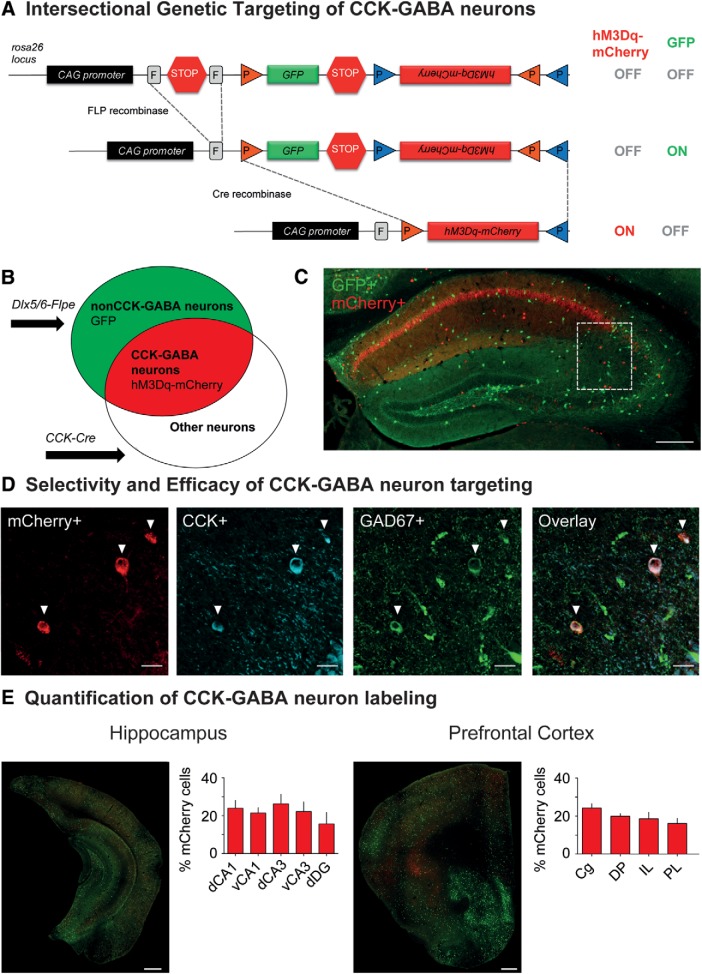
Intersectional genetic approach for targeting hM3Dq receptors to CCK-GABA neurons. ***A***, top, The dual recombinase-responsive *RC::FL-hM3Dq* allele is knocked-in to the Gt(ROSA)26Sor (R26) locus with CAG (chicken β-actin and CMV enhancer) promoter elements. Middle, Flpe-mediated excision of the FRT (rectangles, denoted by *F*)-flanked stop cassette (STOP) permits expression of GFP. Transcription of the mCherry-hM3Dq allele is prevented by the inverted orientation of the sequence and second stop cassette. Bottom, Subsequent Cre-mediated recombination of loxP (orange triangles) and lox2272 (blue triangles) sites constituting the Cre-dependent FLEX switch leads to the removal of the GFP sequences and second stop cassette as well as inversion of the hM3Dq-mCherry sequences into the proper orientation for transcription. Cells expressing Cre and Flpe therefore express hM3Dq-mCherry but not GFP. ***B***, Venn diagrams illustrating intersectional and subtractive cell populations targeted using the intersectional genetic approach. The *Dlx5/6-Flpe* allele is specific to GABAergic cells in the forebrain, whereas the *CCK-Cre* allele is specific to Cre-expressing cells. In mice inheriting all three alleles (*CCK-Cre;Dlx5/6-Flpe;RC::FL-hM3Dq*), cells expressing both Cre and Flpe alleles (i.e., intersectional population) represent CCK-GABA neurons and express hM3Dq-mCherry. Cells expressing only the Flpe allele (i.e., subtractive population) represent nonCCK-GABA neurons and express only GFP. ***C***, Low-magnification images showing hM3Dq-mCherry+ and GFP+ neurons in the dorsal hippocampus of CCK-GABA/hM3Dq+ mice. Scale bar = 250 µm. ***D***, Confocal images of the CA1 SR in CCK-GABA/hM3Dq^+^ mice. mCherry+ cells are labeled in red, CCK^+^ cells are labeled in blue, and GAD67+ cells are labeled in green. Cells expressing all three markers (mCherry, CCK, and GAD67) represent likely CCK-GABA neurons. Scale bar = 25 µm. ***E***, Quantification of hM3Dq-mCherry+ and GFP+ cells in the hippocampus (*n* = 4) and prefrontal cortex (*n* = 5) of CCK-GABA/hM3Dq^+^ mice. Shown is the percentage of hM3Dq-mCherry+ cells out of all labeled cells. Scale bar = 500 µm. dCA1 = dorsal CA1, vCA1 = ventral CA1, dCA3 = dorsal CA3, vCA3 = ventral CA3, dDG = dentate gyrus, Cg = cingulate, DP = dorsal peduncular cortex, IL = infralimbic cortex, PL = prelimbic cortex. All figures present data as mean ± SEM.

To selectively target CCK-GABA neurons, we employed a dual recombinase-based (Cre and Flpe) intersectional approach (Awatramani et al., 2003; Dymecki and Kim, 2007; Taniguchi et al., 2011) where neuronal subtypes are defined by overlapping expression of two genetic markers. A dual recombinase-responsive allele, *RC::FL-hM3Dq* (Sciolino et al., 2016), was combined with Cre and Flpe recombinase alleles, each driven by different promoters: Cre by the CCK (CCKergic) promoter and Flpe by the Dlx5/6 (forebrain GABAergic) intergenic enhancer (Miyoshi et al., 2010). Mice that inherit all three alleles (Cre, Flpe and hM3Dq) were termed CCK-GABA/hM3Dq^+^ mice whereas mice inheriting only two alleles (Cre and hM3Dq) were termed CCK-GABA/hM3Dq^-^ mice. In CCK-GABA/hM3Dq^+^ mice, neurons expressing Cre and Flpe (i.e., CCK-GABA neurons) express the excitatory Gq-coupled receptor hM3Dq fused to the reporter protein mCherry (Fig. 1*A*). In contrast, neurons expressing only Flpe (i.e., nonCCK-GABA neurons) express the reporter protein GFP but not hM3Dq receptors. CCK-GABA/hM3Dq- mice do not express any reporter proteins or hM3Dq receptors (Fig. 1*B*).

To demonstrate that the intersectional approach permitted selective access to CCK-GABA neurons, we quantified the expression of molecular markers consistent with CCK-GABA neuron identity in brain sections from CCK-GABA/hM3Dq^+^ mice. Histologic examination confirmed an abundance of mCherry-positive cells in the stratum radiatum (SR), the hippocampal layer where CCK-GABA neurons are most numerous (Fig. 1*C*; Whissell et al., 2015). The majority of mCherry positive neurons showed immunoreactivity for CCK (79.0 ± 3.2%, *n* = 11; Fig. 1*D*; absolute cell counts are shown in Table 1), a finding which verified the selectivity of CCK-GABA neuron targeting. Next, we evaluated the efficacy of the intersectional approach by determining the proportion of CCK-GABA neurons positive for mCherry expression. CCK-GABA neurons in CCK-GABA/hM3Dq^+^ brain sections were operationally defined as cells with immunoreactivity for the molecular markers CCK and GAD67. Analysis showed that of all neurons showing immunoreactivity for CCK and GAD67, the majority were also immunoreactive for mCherry (73.6 ± 4.6%, *n* = 11; Fig. 1*D*; Table 1). To verify that the GABA neuron labeling in our mouse line was comparable to that in other intersectional models (Whissell et al., 2015), we quantified mCherry+ neurons (likely CCK-GABA neurons) and GFP+ neurons (nonCCK-GABA neurons) in the hippocampus and prefrontal cortex. Consistent with past reports, we observed that CCK-GABA neurons accounted for ∼22% of GABA neurons in the hippocampal subregions and 19% of GABA neurons in prefrontal cortex subregions (Fig. 1*E*; Table 1). Together, these data indicate that the CCK-GABA/hM3Dq line targets CCK-GABA neurons with relatively high selectivity and efficacy.

**Table 1.** Absolute cell counts of mCherry-labeled and GFP-labeled cells in the hippocampus and prefrontal cortex of hM3Dq+ mice

			mCherry-labeled cells		GFP-labeled cells	
			Absolute count		Absolute count	
Main region	Subregion		Mean	SEM	Mean	SEM
Hippocampus	dCA1	CA1, dorsal	31.4	8.8	94.8	15.5
	vCA1	CA1, ventral	22.9	3.2	87.0	19.5
	dCA3	CA3, dorsal	16.9	2.1	48.7	8.4
	vCA3	CA3, ventral	15.9	1.8	56.2	4.2
	dDG	Dentate gyrus, dorsal	15.1	6.7	73.7	17.0
Frontal cortex	Cg	Cingulate	18.6	0.4	57.6	0.4
	DP	Dorsal penducular	8.4	0.1	37.8	0.6
	IL	Infralimbic	10.8	0.4	56.6	0.9
	PL	Prelimbic	23.8	0.3	133.3	1.4

mCherry-labeled cells represent probable CCK-GABA neurons whereas GFP-labeled cells represent nonCCK-GABA neurons.

### Selective chemogenetic activation of CCK-GABA neurons in the hippocampus increases inhibition at CA1 pyramidal neurons

To verify that our model permitted selective activation of CCK-GABA neurons but not other cells, we conducted *in vitro* slice electrophysiology experiments in CCK-GABA/hM3Dq^+^ and CCK-GABA/hM3Dq^-^ mice. All experiments were performed in the SR layer of the hippocampus, where there is a relative abundance of CCK-GABA neurons available for whole-cell patch clamp (Whissell et al., 2015).

**Figure 2.**
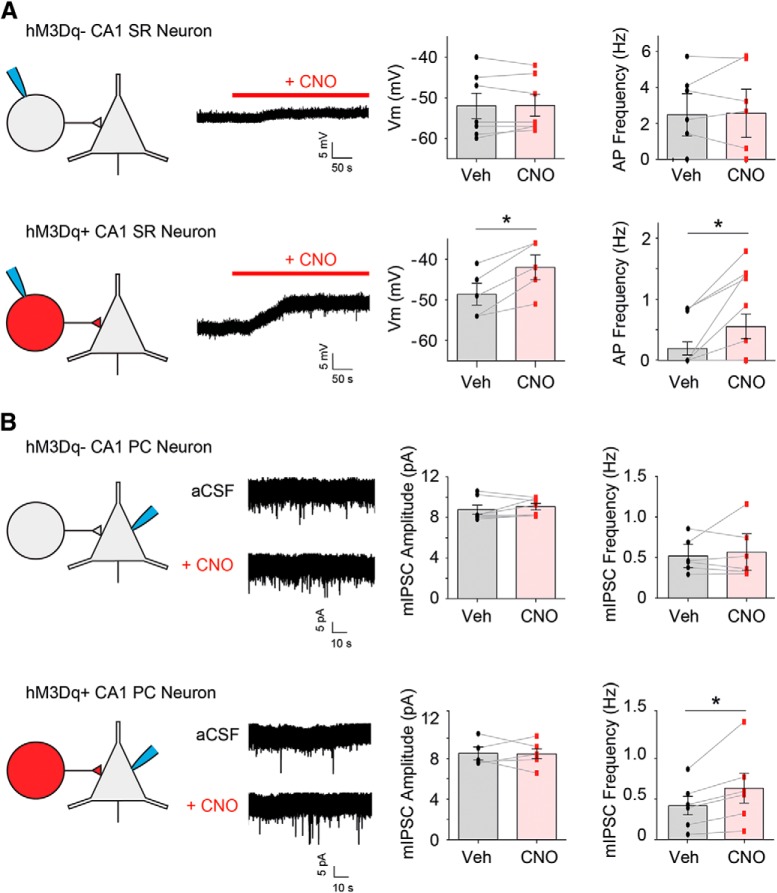
Selective activation of CCK-GABA neurons increases inhibition of CA1 pyramidal neurons in CCK-GABA/hM3Dq mice. ***A***, Whole-cell patch-clamp recordings of CA1 SR neurons in CCK-GABA/hM3Dq^-^ (top) and CCK-GABA/hM3Dq^+^ mice (bottom) with and without CNO. In SR neurons from CCK-GABA/hM3Dq^+^ mice only, the administration of CNO (5 µM) significantly depolarized the membrane potential (*n* = 5) and increased firing rate (*n* = 13). ***B***, Whole-cell patch-clamp recordings from CA1 pyramidal cells (PCs) in CCK-GABA/hM3Dq^-^ (top) and CCK-GABA/hM3Dq^+^ mice (bottom) with and without CNO. In CA1 PC neurons from CCK-GABA/hM3Dq^+^ only (*n* = 6), CNO increased the frequency but not amplitude of mIPSCs. All figures present data as mean ± SEM. The asterisk indicates statistical significance at the *p* < 0.05 level.

To determine whether CCK-GABA neurons could be reliably excited in our model, we examined the response of putative CCK-GABA neurons to CNO, a compound that selectively activates transgenic hM3Dq receptors (Armbruster et al., 2007). It was expected that CNO (5 µM) would depolarize SR cells in CCK-GABA/hM3Dq^+^ but not CCK-GABA/hM3Dq^-^ mice. In CCK-GABA/hM3Dq^+^ mice, we targeted mCherry+ neurons for whole-cell patch clamp (Fig. 2*A*) as our histologic data indicated that the majority of these cells expressed markers consistent with CCK-GABA neuron identity (Fig. 1*D*). Consistent with expectations, CNO significantly depolarized mCherry+ neurons (*t*_(4)_ = 5.66, *p* = 0.0024) and increased their firing frequency (W = 45, *p* = 0.0039). In contrast, CNO did not affect membrane potential or firing rate in mCherry- neurons from CCK-GABA/hM3Dq^-^ mice (all *p*s > 0.40).

We next verified that activation of hippocampal CCK-GABA neurons increased inhibition of their postsynaptic targets, CA1 pyramidal neurons (Basu et al., 2013). CA1 pyramidal neurons were patched and mIPSCs were measured before and after CNO administration (Fig. 2*B*). In CCK-GABA/hM3Dq^+^ mice, CNO significantly increased the frequency (*t*_(5)_ = 2.82, *p* = 0.037) but not amplitude (*p* > 0.92) of mIPSCs in CA1 pyramidal neurons. In contrast, no effects of CNO on mIPSC frequency or amplitude were observed in CA1 pyramidal neurons from CCK-GABA/hM3Dq^-^ mice (all *p*s > 0.37). Collectively, these findings indicate that CNO selectively increases the excitability of putative CCK-GABA neurons and inhibition of CA1 pyramidal neurons in CCK-GABA/hM3Dq^+^ mice.

### Systemic activation of CCK-GABA neurons minimally affects anxiety but enhances memory

**Figure 3.**
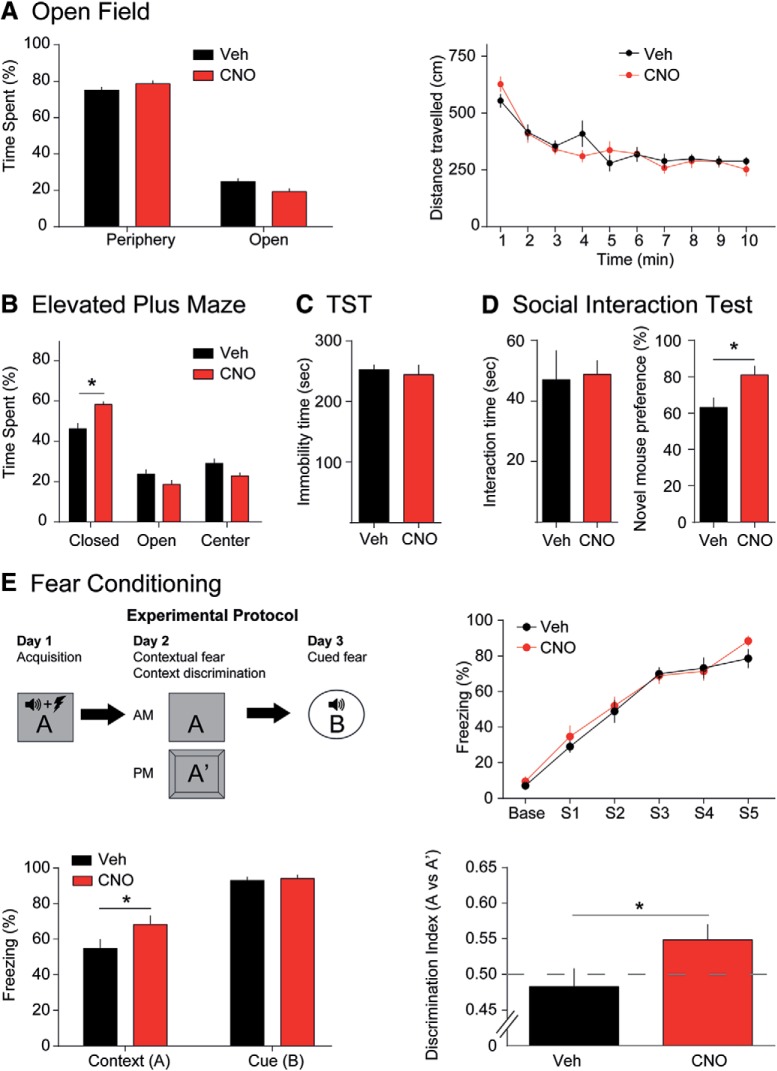
CCK-GABA neuron activation minimally affects overall emotional behavior but enhances contextual fear memory. ***A***, OF test. CNO-treated mice (red, *n* = 10) do not differ from vehicle-treated mice (black, *n* = 9) in center time, periphery time, or distance traveled. ***B***, EPM. CNO-treated mice (*n* = 13) show significantly higher closed arm time than vehicle-treated controls (*n* = 14). However, open and center arm time was not significantly different between groups. ***C***, TS test. Immobility time, an indicator of behavioral despair and depression-like behavior, does not differ between vehicle-treated (*n* = 6) and CNO-treated animals (*n* = 7). ***D***, Social interaction test. Preference for novel mice, an indicator of social recognition, is higher in CNO-treated animals (*n* = 10) than vehicle controls (*n* = 10). Interaction time is unaffected by CNO. ***E***, Fear conditioning and contextual discrimination. Top, left, Experimental protocol. Top, right, CNO-treated (*n* = 13) and vehicle-treated (*n* = 11) animals show similar responses during training sessions. Bottom, left, Recall of contextual but not cued fear conditioning, as evidenced by percentage freezing, is significantly greater in CNO-treated mice. Bottom, right, CNO-treated mice show greater contextual discrimination than vehicle-treated mice. All figures present data as mean ± SEM. Behavioral data in hM3Dq- mice is given in Extended Data Figure 3-1. The asterisk indicates statistical significance at the *p* < 0.05 level.

To determine the effects of CCK-GABA neuron activation on emotional behavior, we compared the performance of vehicle- and CNO-treated CCK-GABA/hM3Dq^+^ mice in the OF, EPM, and TS tests (Fig. 3*A–C*). In the OF test, CNO treatment did not change % center time or total distance traveled (all *p*s > 0.07; Fig. 3*A*). Similarly, CNO treatment did not change % open arm time in the EPM (Fig. 3*B*), which is regarded as the most reliable indicator of anxiety-like behavior (Walf and Frye, 2007). However, CNO did increase % closed arm time (two-way interaction of drug × compartment, *F*_(2,50)_ = 7.79, *p* = 0.0011) a finding which may reflect a subtle increase in anxiety. To ensure that this effect of CNO was mediated by hM3Dq receptors (MacLaren et al., 2016; Gomez et al., 2017), we tested the effects of the drug in CCK-GABA/hM3Dq^-^ mice. We did not observe any effect of the drug in these animals (*F*_(2,36)_ = 0.29, *p* = 0.75; Extended Data Fig. 3-1). In the TS test (Fig. 3*C*), a measure of depression-like behavior, CNO had no effect on immobility time (*p* > 0.67; Steru et al., 1985). Together, these results suggest that CCK-GABA neuron activation does not affect locomotion or depression-like behavior but mildly increases anxiety-like behavior in CCK-GABA/hM3Dq^+^ mice.

10.1523/ENEURO.0360-18.2019.f3-1 Extended Data Figure 3-1 Lack of behavioral response to CNO in hM3Dq– mice. ***A***, EPM. CNO-treated mice (*n* = 10) do not differ from Veh-treated controls (*n* = 10) in performance. ***B***, Social interaction test. Preference for novel mice does not differ between CNO-treated animals (*n* = 6) and vehicle controls (*n* = 7). ***C***, Novel object recognition test. CNO-treated animals (*n* = 13) and Veh-treated animals (*n* = 13) did not differ in object recognition (left) or interaction time (right). ***D***, Puzzle box. Escape latencies for CNO-treated (*n* = 5) and Veh-treated mice (*n* = 6) is comparable in all trials. All figures present data as mean ± SEM. Download Figure 3-1, TIF file.

Subtle changes in anxiety may potentially affect other behavioral functions in mice, including social behavior. Accordingly, we next examined the effects of activating CCK-GABA neurons in the three-chambered social interaction test (Moy et al., 2004). In the first portion of this test, a mouse is exposed to a novel conspecific. Interaction time with this novel mouse is deemed to reflect sociability but is constrained by anxiety (File and Hyde, 1978). CNO treatment did not affect interaction time (*p* > 0.86; Fig. 3*D*), a finding which suggests that systemic activation of CCK-GABA cells does not affect sociability or anxiety. In the second phase of the social interaction test, we examined the effects of CNO on social recognition. To demonstrate social recognition, a mouse must exhibit a preference for a novel conspecific over a familiar conspecific (File and Hyde, 1978). Social recognition is constrained by anxiety, but also involves memory processes and is regulated by the hippocampus (Tanimizu et al., 2017), a brain region where CCK-GABA neurons are relatively abundant (Whissell et al., 2015). Surprisingly, we found that CNO enhanced social recognition in CCK-GABA/hM3Dq^+^ mice (*t*_(17)_ = 2.44, *p* = 0.026) but not CCK-GABA/hM3Dq^-^ mice (*t*_(11)_ = 1.98, *p* = 0.078; Extended Data Fig. 3-1). These data suggest that the activation of CCK-GABA neurons enhances social recognition memory in CCK-GABA/hM3Dq^+^ mice without changing overall sociability.

If CCK-GABA neurons facilitated social recognition by enhancing memory processes, then activation of these cells might also improve the performance of other memory tasks. Accordingly, we determined the effects of CCK-GABA neuron activation on performance in the fear conditioning assay (Fig. 3*E*; Phillips and LeDoux, 1992). In this test, the animal learns to associate a stimulus (either a tone or context) with an aversive experience (an electric footshock). After multiple pairings between this stimulus and the aversive experience, the animal acquires a conditioned response (freezing) to the stimulus. This freezing response is termed conditioned fear and is indicative of learning. Given that our earlier results showed an enhancement in social recognition, we expected that conditioned fear in CCK-GABA/hM3Dq^+^ mice would be enhanced by CNO.

During the training session, vehicle- and CNO-treated mice showed similar levels of freezing (*p* > 0.54; Fig. 3*E*). This finding suggested that acquisition of conditioned fear was comparable in both groups. Twenty-four hours after training, mice were given a second CNO injection and re-exposed to the training context to measure contextual fear memory. Interestingly, freezing to the training context was greater in CNO-treated mice (*t*_(22)_ = 1.90, *p* = 0.036), suggesting enhanced contextual fear memory retrieval. The next day (48 h after training), mice were given a third CNO injection and re-exposed to the auditory tone to measure cued fear conditioning. This behavior was not affected by CNO (*t*_(11)_ = 0.39, *p* = 0.35). These results indicated that CCK-GABA neuron activation enhances the retrieval of contextual fear memory.

To exclude the possibility that anxiety influenced the contextual fear memory in CCK-GABA/hM3Dq^+^ mice, we next measured contextual discrimination (Whissell et al., 2013). In this task, mice are exposed to a novel context (A’) that is highly similar to the training context (A) in features. Mice with strong contextual memories show selectively increased freezing in the training context but not the similar context. In contrast, anxious mice typically show high freezing in both contexts due to fear-induced stimulus generalization (Huckleberry et al., 2016). Memory performance in this task is evaluated using the discrimination ratio (*d* = freezing in A/[freezing in A + freezing in A’], ranging from 0 to 1). Contextual discrimination is notoriously difficult; mice may take 9 d or more to learn this behavior (Whissell et al., 2013). Surprisingly, CNO-treated mice exhibited successful context discrimination after 1d of training (*t*_(22)_ = 1.98, *p* = 0.030; Fig. 3*E*). These results support the argument that enhanced fear conditioning with CCK-GABA neuron activation is due to enhanced memory rather than increased anxiety.

### Activation of CCK-GABA neurons enhances novel object recognition

**Figure 4.**
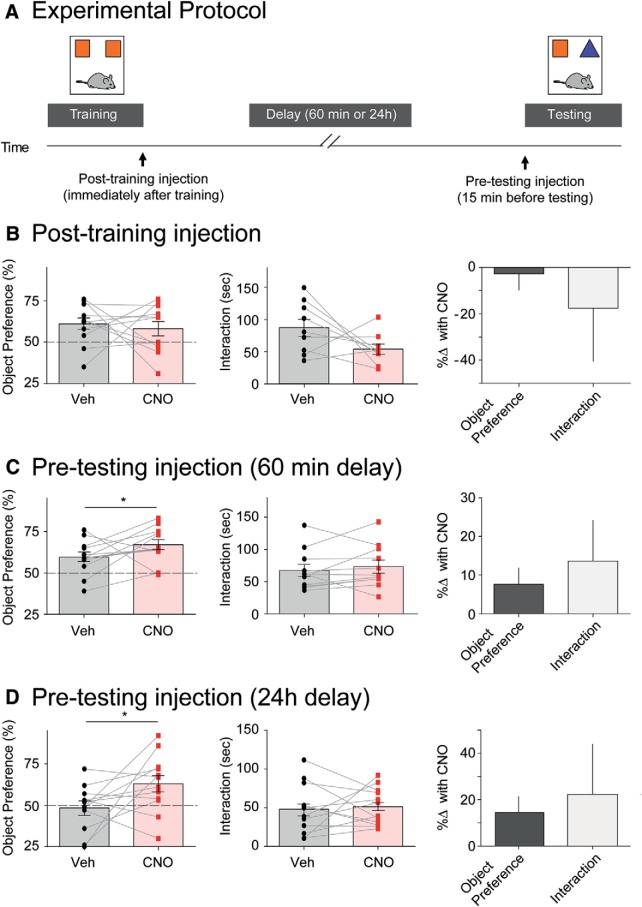
Selective activation of CCK-GABA neurons enhances novel object recognition memory. ***A***, Experimental protocol showing training (15 min), delay (1 or 24 h), and testing (15 min) phases. Post-training injections were given immediately following training, whereas pre-testing injections were given 15 min before testing. ***B***, Post-training CNO injection did not affect novel object preference (*n* = 11) but tended to reduce interaction time. ***C***, In the 1-h protocol, pre-testing CNO injection increased novel object preference (*n* = 12) but did not affect interaction time. ***D***, In the 24-h protocol, pre-testing CNO injection increased novel object preference (*n* = 12) but not affect interaction time. All figures present data as mean ± SEM. Behavioral data in hM3Dq- mice is given in Extended Data Figure 3-1. The asterisk indicates statistical significance at the *p* < 0.05 level.

The above results suggested that CCK-GABA neuron activation is associated with improved memory performance in several tasks. However, it was unclear how CCK-GABA neuron activation bolsters memory performance. Memory processing is argued to be composed of several stages, (acquisition, maintenance/consolidation and retrieval), each of which may be modulated by CCK-GABA neuron activity. To determine if memory maintenance/consolidation or retrieval are modulated by CCK-GABA activity, we examined how CNO injection during these stages affected memory in the novel object recognition task (Fig. 4*A*; Warburton and Brown, 2015).

In this task, the mouse is placed in an arena where it is allowed to interact with two identical objects (training; where object memories are acquired). In the second phase, the mouse is removed from the arena for 1 h (post-training; where memories are maintained/consolidated). In the third phase, the mouse is returned to the arena, which now includes a familiar object from the training phase and a novel object (testing; where object memories are retrieved). Mice that have a strong memory of the familiar object show a preference for the novel object and interact with it more frequently. To discern the effects of CNO on different phases of memory processing, CCK-GABA/hM3Dq^+^ mice were given drug injections in the post-training or pre-testing period. We expected that the pre-testing injection of CNO would enhance object preference in the novel object recognition test, as pre-testing injection of CNO enhanced the retrieval of contextual fear memory in the fear conditioning task.

Post-training injections did not affect novel object preference (*p* > 0.34), but tended to reduce object interaction (*p* = 0.053; Fig. 4*B*). The failure of post-training CNO to enhance object recognition does not support the notion that CCK-GABA neuron activation affects memory maintenance/consolidation. The pre-testing injection was given 15 min before testing, as CNO takes ∼15 min to reach peak levels in plasma (Guettier et al., 2009). In contrast, pre-testing CNO injection significantly increased novel object preference (*t*_(11)_ = 1.78, *p* = 0.05) but did not affect object interaction (*p* > 0.22; Fig. 4*C*). The memory-enhancing effects of pre-testing CNO required the expression of hM3Dq receptors, as CCK-GABA/hM3Dq^-^ mice were unaffected by CNO (*t*_(12)_ = 0.75, *p* = 0.23; Extended Data Fig. 3-1). Collectively, these results suggested that CCK-GABA neuron activation enhances the retrieval of memories acquired 1 h earlier. To verify that CCK-GABA neuron activation also enhanced the retrieval of long-term memories, we investigated the effect of CNO on object recognition measured 24 h after training. Consistent with our previous result, CNO enhanced object recognition measured 24 h after training in CCK-GABA/hM3Dq^+^ mice (*t*_(11)_ = 2.13, *p* = 0.028) but did not affect interaction time (*p* = 0.34; Fig. 4*D*).

### Activation of CCK-GABA neurons enhances performance in the puzzle box test

The above results showed that the activation of CCK-GABA neurons enhanced performance in three different memory assays: social recognition, contextual fear conditioning and novel object recognition (Figs. 3*D*,*E*, 4). However, an interesting question is whether CCK-GABA neuron activation enhances performance in a cognitive task that does not directly depend on memory processes.

**Figure 5.**
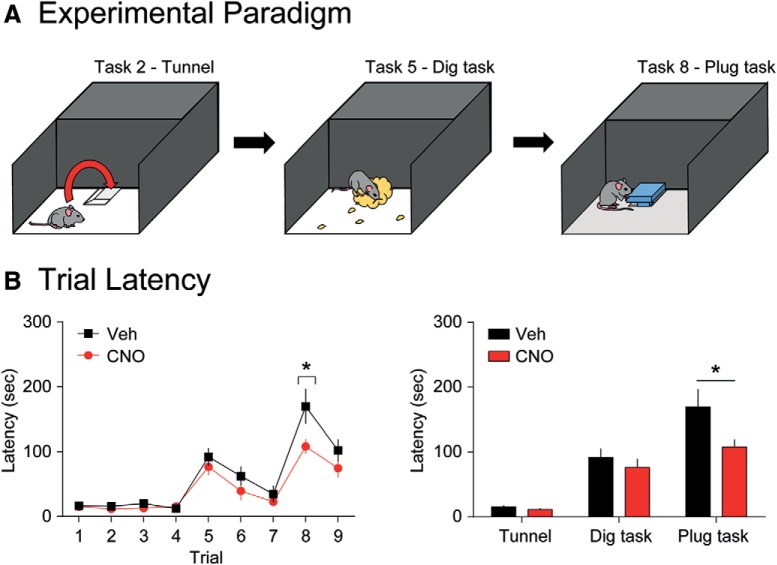
Selective activation of CCK-GABA neurons enhances performance in the puzzle box test. ***A***, Cartoon of the puzzle box test showing all trials in which a new obstacle is introduced (trial 2: underpass; trial 5: dig; trial 8: plug). In these trials, the mouse must acquire a new strategy to escape from the start area into the goal area. ***B***, left, Escape latency of CNO- (*n* = 11) and vehicle-treated (*n* = 14) CCK-GABA/hM3Dq mice in all nine trials of the puzzle box test. Lower escape latencies indicate better performance. CNO-treated mice show significantly lower escape latency only in trial 8 (plug task). ***B***, right, Escape latencies for CNO-treated and Veh-treated mice in all novel obstacle trials (underpass, dig, and plug task). All figures present data as mean ± SEM. Behavioral data in hM3Dq- mice is given in Extended Data Figure 3-1. The asterisk indicates statistical significance at the *p* < 0.05 level.

To determine the effects of CCK-GABA neuron activation on cognitive performance, we subjected CCK-GABA/hM3Dq^+^ mice to the puzzle box test. This assay is regarded as an animal model of executive function, cognitive flexibility and problem solving. The test takes place in a two-chambered apparatus: one chamber is brightly illuminated and exposed to air (“start area”), whereas the other chamber is enclosed by walls and filled with bedding (“goal area”; Fig. 5*A*). The mouse begins in the start area and must navigate to the goal area to complete the task. However, over a series of trials, the path to the goal area is blocked by a series of increasingly difficult obstacles. To reach the goal area, mice must develop strategies to overcome these obstacles. In trial 2, mice must learn to use an underpass (underpass task). In trial 5, the underpass is blocked with bedding and mice must dig through this bedding (dig task). In trial 8, the underpass is blocked with a plug and mice must remove this plug (plug task). As the obstacle in each of these trials (T2, T5, and t8) is novel, a mouse cannot rely on a previously learned strategy (or memory) and must develop a new approach to the problem. If CCK-GABA neuron activation enhanced cognitive function, it is likely that CNO-treated mice would complete trials more quickly than vehicle-treated mice. Indeed, CNO-treated mice generally performed all three tasks more quickly than vehicle-treated mice (main effect of drug, *F*_(1,23)_ = 4.70, *p* = 0.041; Fig. 5*B*). However, *post hoc* analysis indicated that CNO-treated mice only performed significantly better than vehicle-treated controls on the plug task (T8; Fig. 5*B*). This enhanced performance was not due to CNO specifically, as CCK-GABA/hM3Dq^-^ mice did not respond to the drug (*F*_(1,9)_ = 2.74, *p* = 0.13; Extended Data Fig. 3-1). These data support the notion that CCK-GABA neuron activation facilitates general cognitive function as well as memory performance.

## Discussion

In the present study, we used a dual-recombinase strategy to selectively activate CCK-GABA neurons during an array of emotional and cognitive behaviors. Histologic and electrophysiological data showed highly effective targeting and CNO-mediated activation of CCK-GABA cells, respectively. Behavioral data revealed that the activation of CCK-GABA neurons enhances memory and cognitive performance in CCK-GABA/hM3Dq^+^ mice with minimal effects on anxiety. As a recent study showed that CNO is converted to clozapine and clozapine can affect the brain without binding to hM3Dq receptors (Gomez et al., 2017), we also investigated whether CNO had effects that were not dependent on hM3Dq receptors. As we did not observe any effects of CNO in CCK-GABA/hM3Dq^-^ mice, it is most likely that our behavioral effects are mediated by the activation of hM3Dq receptors expressed in CCK-GABA cells.

To our knowledge, this is the first report which directly reveals a selective contribution of CCK-GABA neuron activity to enhancement of cognitive performance. How might CCK-GABA neuron activation facilitate cognition and memory? The signaling by CCK-GABA neurons is complex as it involves both the inhibitory neurotransmitter GABA and the neuropeptide CCK, which tends to be excitatory (Lee et al., 2011). In the hippocampus, the release of GABA from CCK-GABA neurons inhibits CA1 pyramidal cells (Basu et al., 2013) whereas the release of CCK excites parvalbumin-expressing GABA neurons (Lee et al., 2011). As parvalbumin-expressing GABA neurons also inhibit CA1 pyramidal cells, both these effects may ultimately increase inhibitory tone within the hippocampus. Several studies have demonstrated that inhibitory tone supports hippocampus-dependent memory processes, including contextual discrimination (Whissell et al., 2013; Engin et al., 2015), a behavior which was increased by CCK-GABA neuron activation in the present study. Contextual discrimination is thought to require the orthogonal representation of contextual memories in non-overlapping populations of neurons (Aimone et al., 2011). By increasing inhibition, CCK-GABA neurons may effectively reduce “noise” in the hippocampus, making it possible to encode orthogonal memories that are easily discriminated. Through their inhibitory actions in the hippocampus, CCK-GABA neurons may play a role in “signal amplification” by enhancing the signal-to-noise ratio at the network level (Bartos and Elgueta, 2012).

Additionally, CCK-GABA neurons may contribute to memory processes via effects on other forms of network processing. Most notably, CCK-GABA neurons may contribute to the generation of theta oscillations, which are linked to cognition and memory (Curley and Lewis, 2012). Inhibition of a GABAergic cell population which includes CCK-GABA neurons impairs theta oscillations in the cortex and hippocampus (Nagode et al., 2014; Nguyen et al., 2014), an effect associated with impaired memory (Raver and Keller, 2014). Accordingly, several of the behavioral effects shown here may be explained by CCK-GABA neuron-mediated sculpting of cortical oscillations. As we did not examine cortical oscillations here, this exciting possibility may be addressed in future studies.

Our model, which permits CCK-GABA neuron activation via hM3Dq receptors, does not allow for the specific control of CCK or GABA release from these cells. However, it is most likely that CCK and GABA neurotransmission jointly contribute to our behavioral effects. Most notably, the cluster of behaviors affected in this study differs considerably from those previously linked to GABAergic transmission in CCK-GABA neurons (Brown et al., 2014; Schmidt et al., 2014). In one transgenic model, GABA synthesis was inhibited in CB1 receptor-expressing cells (Brown et al., 2014) the majority of which are CCK-GABA neurons (Marsicano and Lutz, 1999). In contrast to our findings, this manipulation enhanced cued fear but did not affect contextual fear learning (Brown et al., 2014). In another report, inhibition of GABA synthesis in CCK-expressing cells disrupted olfaction and locomotion but not recognition memory or anxiety (Schmidt et al., 2014). While these models and our own are different in the extent and direction to which they manipulate GABA release (Brown et al., 2014; Schmidt et al., 2014), they illustrate that GABA release accommodates only part of CCK-GABA neuron functionality. Notably, CCK release from CCK-GABA neurons may be linked to the subtle increase in anxiety-like behaviors observed in our study and other models (Bowers and Ressler, 2015). As CCK and GABA signaling interact *in vivo* (Lee et al., 2011) it may be impractical to separate them experimentally.

An important consideration is that the CCK-GABA/hM3Dq model used here permits only global activation of CCK-GABA neurons. As CNO was injected in the intraperitoneal cavity and processed systemically, it activates CCK-GABA neurons across the entire forebrain. Although multiple CCK-GABA neuron populations may contribute to the behaviors studied here, we propose that CCK-GABA neurons in the hippocampus play an important role. Importantly, our electrophysiological data shows that CCK-GABA neuron activation is associated with increased inhibition of CA1 pyramidal neurons in the hippocampus. Many behaviors enhanced by CCK-GABA neuron activation (including contextual discrimination, contextual fear conditioning and puzzle box performance) are regulated by the hippocampus (Phillips and LeDoux, 1992; Frankland et al., 1998; Ben Abdallah et al., 2011). CNO-mediated enhancement of performance in these tasks may arise from altered hippocampal network behavior, as a recent study found that disruption of CCK-GABA neuron wiring in the hippocampus was associated with abnormal neuronal oscillations, dysregulated spatial memory encoding and impaired learning (Del Pino et al., 2017). Similarly, others have shown that indirect inhibition of CCK-GABA neurons in the CA1 region altered contextual fear conditioning and object recognition (Basu et al., 2016). Although hippocampal CCK-GABA neurons are likely involved in our behavioral effects, it is also possible that other CCK-GABA populations contribute. CCK-GABA neurons in the medial prefrontal and perirhinal cortex (Whissell et al., 2015) may be particularly relevant to the enhanced recognition memory observed in CNO-treated CCK-GABA/hM3Dq mice, as recognition memory depends on these areas (Warburton and Brown, 2015). Ultimately, functional characterization of individual populations of CCK-GABA neurons will require a comprehensive, region-specific approach. In this regard, selective delivery of CNO to individual brain regions through cannulation may be productive.

Several disorders may involve disruption of CCK-GABA neuron signaling, including chronic stress (Reich et al., 2013) and schizophrenia (Curley and Lewis, 2012). In the case of schizophrenia, evidence suggests that dysregulated CCK-GABA neuron signaling may contribute to the pathogenesis of the disorder (Curley and Lewis, 2012; Nguyen et al., 2014). In human studies of schizophrenia and animal models of the disorder, there is reduced expression of CCK and CB1 receptor markers (Eggan et al., 2008; Hashimoto et al., 2008) as well as suppression of the cortical rhythms mediated by CCK-GABA neurons (Raver and Keller, 2014). Our data support a role for the dysregulation of CCK-GABA activity in cognitive dysfunction associated with these disorders. If disrupted CCK-GABA neuron signaling contributes to the pathogenesis of schizophrenia, then activation of CCK-GABA neurons might prove therapeutic. The CCK-GABA/hM3Dq model provides a useful strategy for testing this possibility.

## Synthesis

Reviewing Editor: Thomas McHugh, RIKEN Center for Brain Science Decisions are customarily a result of the Reviewing Editor and the peer reviewers coming together and discussing their recommendations until a consensus is reached. When revisions are invited, a fact-based synthesis statement explaining their decision and outlining what is needed to prepare a revision will be listed below. The following reviewer(s) agreed to reveal their identity: Susan Ferguson.

The reviewers find the manuscript to be much improved, stating it provides new insight into the role of CCK-GABA neurons in cognition and memory. They are in agreement that the new data, additional analysis and clarified methods have addressed their previous concerns and have no further requests prior to publication.
