# Supplementary figures and images for: Selective Activation of Cholecystokinin-Expressing GABA (CCK-GABA) Neurons Enhances Memory and Cognition
Source: eNeuro. 2019 Feb 27;6(1):ENEURO.0360-18.2019. doi: 10.1523/ENEURO.0360-18.2019 (PMC6397954; doi:10.1523/ENEURO.0360-18.2019)

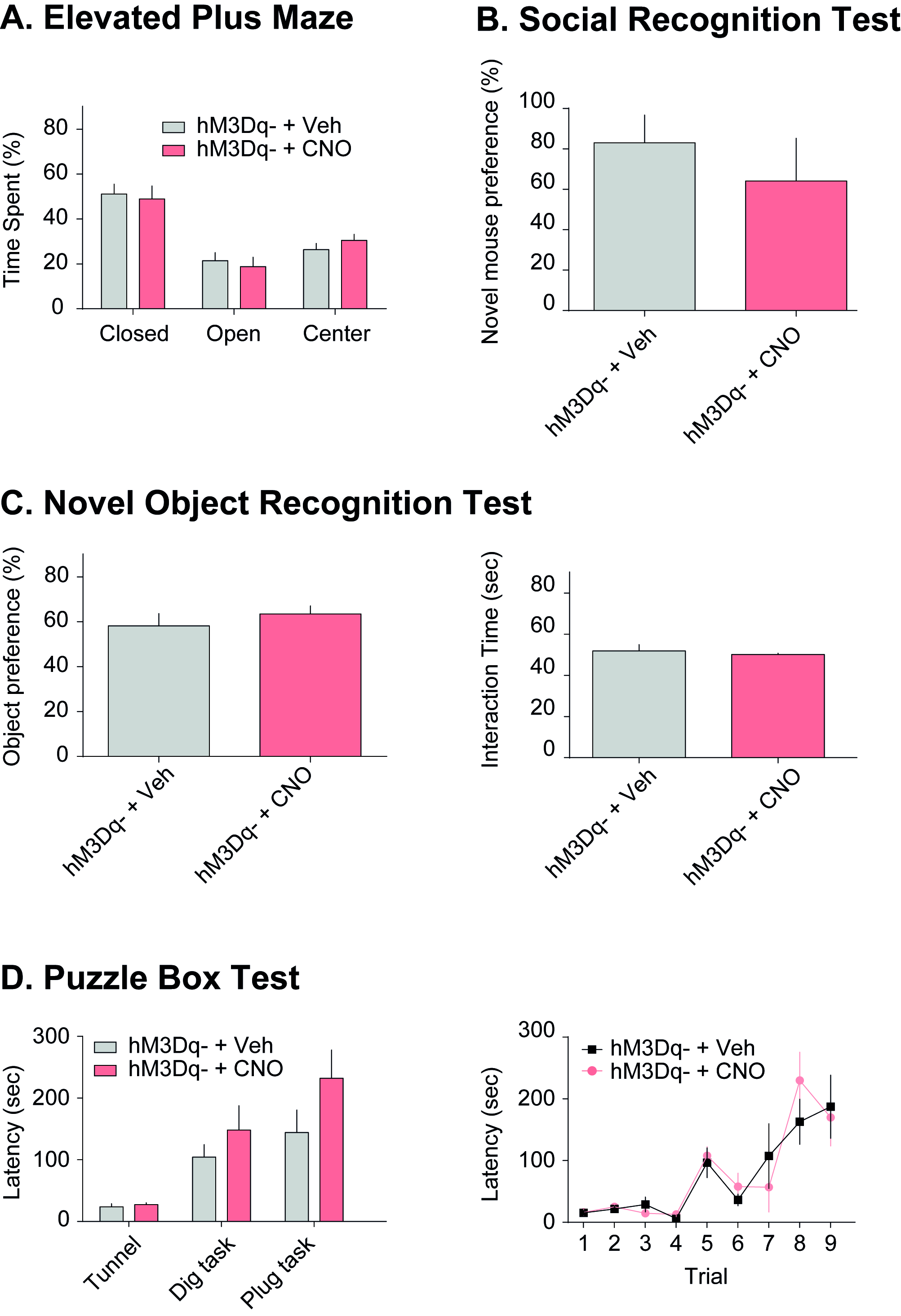

Supplement: Extended Data Figure 3-1 — Lack of behavioral response to CNO in hM3Dq– mice. A, EPM. CNO-treated mice (n = 10) do not differ from Veh-treated controls (n = 10) in performance. B, Social interaction test. Preference for novel mice does not differ between CNO-treated animals (n = 6) and vehicle controls (n = 7). C, Novel object recognition test. CNO-treated animals (n = 13) and Veh-treated animals (n = 13) did not differ in object recognition (left) or interaction time (right). D, Puzzle box. Escape latencies for CNO-treated (n = 5) and Veh-treated mice (n = 6) is comparable in all trials. All figures present data as mean ± SEM. Download Figure 3-1, TIF file. [file sup_enu-eN-NWR-0360-18-s02.tif]
